# VK210 and VK247 genotypes of *Plasmodium vivax* in anopheline mosquitoes from Brazilian Amazon

**DOI:** 10.1038/s41598-019-45809-5

**Published:** 2019-06-28

**Authors:** Erian de Almeida Santos, Izis Mônica Carvalho Sucupira, Bruno Matheus de Oliveira Martins, Ricardo José de Paula Souza e Guimarães, Clístenes Pamplona Catete, Raimundo Tadeu Lessa de Souza, Ana Cecília Feio dos Santos, Marinete Marins Póvoa

**Affiliations:** 1 Federal University of Pará, Pós Graduação em Biologia de Agentes Infecciosos e Parasitários, Belém, Pará 66075-110 Brazil; 2 Evandro Chagas Institute, Laboratory of Basic Research in Malaria - Entomology, Parasitology Section, Ananindeua, Pará 67030-000 Brazil; 3 Evandro Chagas Institute, Laboratory of Geoprocessing, Ananindeua, Pará 67030-000 Brazil

**Keywords:** Parasite evolution, Malaria

## Abstract

*Plasmodium vivax* sporozoites are differenced by circumsporozoite protein. Studies on the circulation of *P. vivax* VK210 and *P. vivax* VK247 in anopheline mosquitoes are important to verify the adaptability of these parasites on mosquitoes in different locations and periods. This study aimed to describe and compare the distribution of these genotypes in anopheline mosquitoes from four states of the Brazilian Amazon. Epidemiological databases about CSP infections on mosquitoes from Pará (2000–2015), Amapá (2000–2010), Roraima (2000–2003 and 2009–2011) and Acre States (2012–2015) were used for analysis. A total of 895 specimens were found infected mainly by *P. vivax* VK210. We showed that the distribution of *P. vivax* VK247 changed over time in the main malaria vectors on the Brazilian Amazon. We note that *A. darlingi* was abundant in certain localities while *A. albitarsis* s.l. in anothers, which highlights the importance of entomological studies for the control of human malaria.

## Introduction

More than 200 million cases of malaria, an acute disease caused by a protozoon of the genus *Plasmodium* and transmitted by anopheline mosquitoes in tropical and subtropical areas, occurred worldwide in 2018^1^. In Brazil, almost all malaria cases are present in the Amazon Region (99.5%)^2^, which is comprised by nine states: Acre, Amapá, Amazonas, Rondônia, Roraima, Pará, Maranhão, Mato Grosso, and Tocantins. Among these states, Amazonas presented the highest malaria incidence in 2017 (41.94%), followed by Acre (19.01%) and Pará (18.91%)^3,4^.

The main malaria vectors in the Brazilian Amazon are *Anopheles (Nyssorhynchus) darlingi* Root 1926, *A. (Nys.) aquasalis* Curry 1932, and *A. (Nys.) albitarsis* species Lynch-Arribálzaga, 1878^5,6^, together with some other secondary vectors in transmission^6,7^. Malaria sporozoites, the mosquito-infecting stage, have a circumsporozoite protein (CSP) that exhibits variations in the central domain of the *Plasmodium vivax* gene, characterizing variants as: VK210, VK247, and *P. vivax*-like^8–10^. Previous studies have detected the natural infection by *P. vivax* genotypes in anopheline mosquitoes in the Brazilian Amazon^7,11–13^ and experimentally it was shown that some species are susceptible to infection by *P. vivax* VK210 and VK247^14,15^. Anopheline infection with specific plasmodia genotypes may differ between different geographical areas^16^.

Studies that verify the main transmitters of the disease, as well as to investigate the circulation of *P. vivax* CSP variants in the Brazilian Amazon can provide relevant information about the main vectors involved in disease transmission and the adaptation of these parasites in different locations and periods. We set out to describe and compare the distribution of CSP genotypes of *P. vivax* VK210 and VK247 in anopheline mosquitoes from four Brazilian Amazon states from 2000 to 2015.

## Results

### Mosquito abundance and distribution

A total of 83,511 anopheline mosquitoes were collected during the study period, including the species *A. albitarsis* s.l. (41.12%), *A. darlingi* (38.09%)*, A. triannulatus* s.l. (5.89%), *A. nuneztovari* s.l. (4.65%)*, A. braziliensis* (3.56%)*, A. strodei* (1.63%)*, A. minor* (1.05%), *A. peryassui* (0.93%)*, A. oswaldoi* (0.85%)*, A. aquasalis* (0.68%), and other species at very low frequency.

The Human Biting Rate (HBR) values were higher among *A. darlingi* species, mainly in the states of Pará and Acre (ranging from 0.04 to 21.46 bites/per person/year) and *A. albitarsis* s.l. in Amapá and Roraima (0.01 to 16.86 bites/per person/year). While the higher Sporozoite Rate (SR) were detected mainly in the species *A. darlingi* (ranging 0.38 to 5.60) in all analyzed States (see more in Supplementary Table S2).

### Distribution of CSP genotypes of *P. vivax* in *Anopheles mosquitoes*

It was detected 895 anopheline mosquitoes naturally infected with *P. vivax* genotypes, 446 in *A. albitarsis* s.l. (49.83%), 404 in *A. darlingi* (45.13%), 21 in *A. nuneztovari* s.l. (2.35%), 11 in *A. braziliensis* (1.23%), six in *A. aquasalis* (0.67%), and three in *A. triannulatus* s.l. (0.33%).

**Figure 1:**
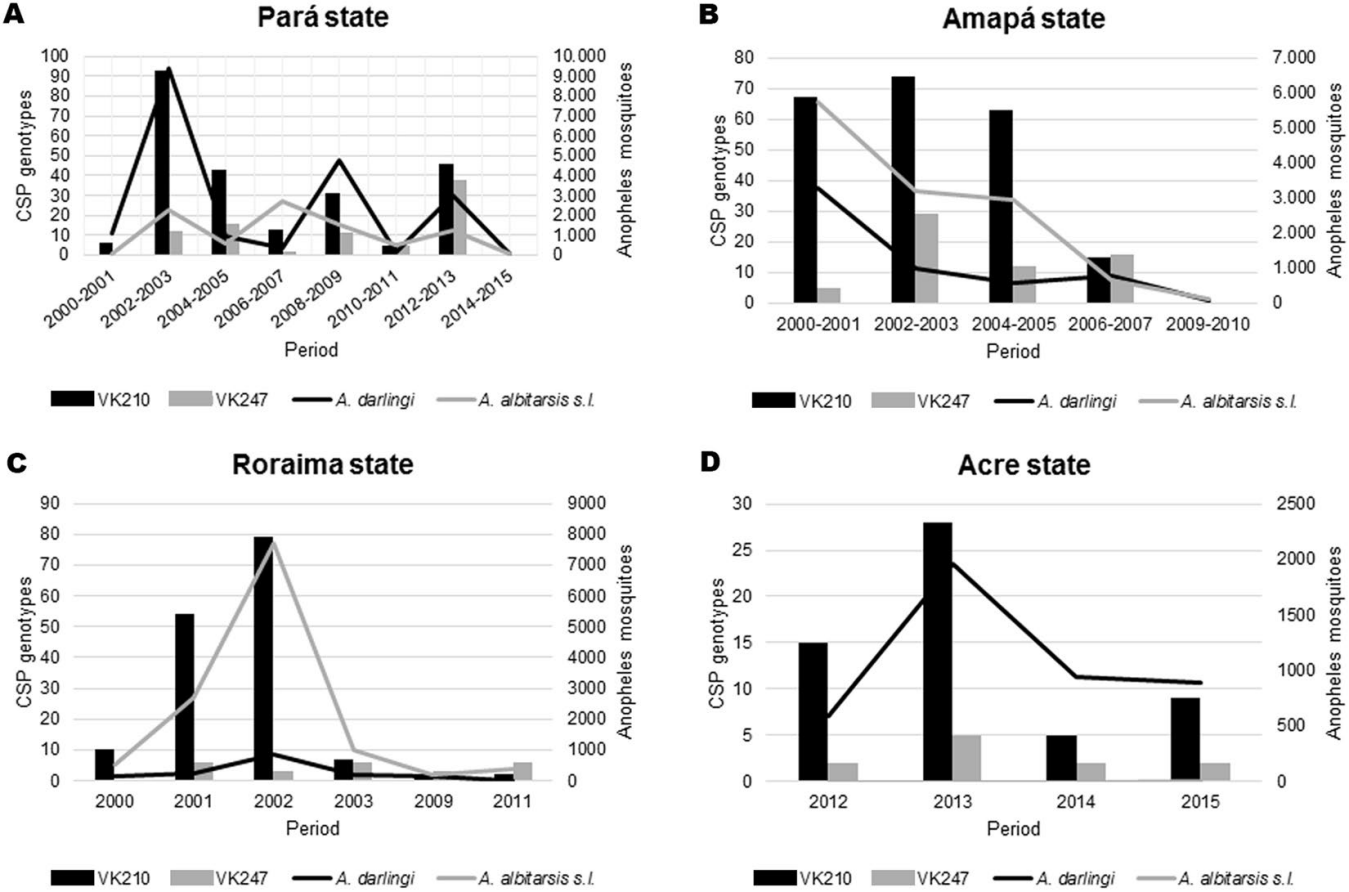
Frequency of *P. vivax* CSP genotypes and abundance of *Anopheles darlingi* and *A. albitarsis* s.l. in (**A**) Pará, (**B**) Amapá, (**C**) Roraima and (**D**) Acre states, 2000 to 2015.

The frequency of mixed infections was very low (0.44%), two infections of *P. vivax* VK210/VK247 in *A. darlingi* (0.22%) and one in *A. triannulatus* s.l. (0.11%) from Pará state, and one infection of *P. vivax* VK210/*P. falciparum* in *A. albitarsis* s.l. (0.11%) from Roraima. *P. vivax* genotype VK210 was widely distributed and more prevalent than *P. vivax* VK247, and *A. darlingi* and *A. albitarsis* s.l. had the highest number of infected specimens in all analyzed States (Fig. 1).

In Pará State, the VK210 and VK247 infections in *A. darlingi* were significative on the years from 2002 to 2007 (p < 0.05), but non significative (p > 0.05) from 2008 onwards. On the other hand, in Roraima State, only VK247 infection in *A. albitarsis* s.l was significative in two periods, 2002–2003 (p = 0.0315) and 2009–2011 (p = 0.0533). Despite of the higher abundance and number of infection of *A. albitarsis* s.l. in Amapá and Roraima States, the *A. darlingi* mosquitoes showed the highest SR in some analyzed periods (Table 1).

**Table 1:** Number of CSP infection and SR in the main malaria vectors of Brazilian Amazon.

State/Period	*A. darlingi*	*A. albitarsis s.l.*	p-value	*A. darlingi*	*A. albitarsis s.l.*	p-value
	VK210 (SR %)			VK247 (SR %)		
**Pará**						
2000–2001	6 (0.56)	—	NA	—	—	NA
2002–2003	62 (0.66)	31 (1.39)	0.0005	7 (0.07)	5 (0.22)	0.0486
2004–2005	36 (3.89)	7 (1.25)	0.0032	15 (1.62)	1 (0.18)	0.0089
2006–2007	6 (1.72)	7 (0.26)	<0.0001	2 (0.57)	—	NA
2008–2009	25 (0.53)	6 (0.57)	0.8615	11 (0.23)	—	NA
2010–2011	2 (1.45)	3 (0.56)	0.2815	2 (1.45)	3 (0.56)	0.2815
2012–2013	46 (1.52)	—	NA	23 (0.76)	15 (1.22)	0.1454
**Amapá**						
2000–2001	14 (0.43)	53 (0.92)	0.0080	—	5 (0.09)	NA
2002–2003	22 (2.20)	52 (1.63)	0.2317	2 (0.20)	27 (0.84)	0.0315
2004–2005	9 (1.61)	54 (1.82)	0.7229	2 (0.36)	10 (0.34)	0.9424
2006–2007	5 (2.20)	9 (2.62)	0.6028	7 (3.08)	9 (2.62)	0.7449
**Roraima**						
2000–2001	11 (2.92)	53 (1.64)	0.0744	—	6 (0.18)	NA
2002–2003	15 (1.40)	71 (0.82)	0.0531	3 (0.28)	6 (0.07)	0.0315
2009–2011	1 (0.80)	2 (0.34)	0.4735	1 (0.80)	8 (1.37)	0.0533

Values that were not possible for analysis in the Binomial test were considered as not applicable (NA).

## Discussion

The maintenance of human malaria cases in Brazil has been the result of presence of areas favorable to the development of *Anopheles* mosquitoes in the Amazon. Here, our study provided entomological and epidemiological data about the distribution profile of *P. vivax* variants in *Anopheles* species, which is relevant for the knowledge of the main transmitters of the malaria parasites in different endemic sites that are distinguished by geographical, socioeconomic, and cultural characteristics^17^.

Some areas in Acre state may present different degrees of deforestation, which can influence the malaria transmitter density. In the municipality of Acrelândia (AC), 748 km far from Cruzeiro do Sul, the influence of seasonality and human action in natural environments lead to an increase in the *A. darlingi* density^18^. If these factors occur in Cruzeiro do Sul, there is a possibility of the creation of more breeding sites of this species in the region.

The municipality of Cruzeiro do Sul (AC) has potential to be a hotspot zone for cases of human malaria due to practically all the mosquitoes found belong to *A. darlingi* species. Remote sensing analyses of agricultural settlements in the Brazilian Amazon found hotspots with >80% positivity for *A. darlingi* larvae, showing the potential for transmission in residents within 400 m of these areas^19^. In addition, the large number of water collections near residences and the economic incentive promoted by the state government of Acre to create tanks for fish farming^17^ can promote an increase in the *A. darlingi* larvae hotspots and consequently maintain the endemicity in the region.

Besides Acre state, *A. darlingi* was also abundant in Pará, while *A. albitarsis* s.l. in Amapá and Roraima state. Furthermore, the highest HBR indexes were in *A. darlingi* and *A. albitarsis* s.l,.which show the epidemiological importance of these vectors in endemic localities. In Brazilian Amazon, these two species presented high degree of anthropophilia as demonstrated in a study conducted in Roraima and Rondônia states when compared to other anopheline species^20^.

In addition, at certain periods, in Pará and Amapá, the SR of *A. darlingi* was higher than *A. albitarsis* s.l., even when its density was lower. This highlights a concern of public health for this species that can maintain the disease transmission even when they are at low frequency^21,22^.

In Cruzeiro do Sul (AC) the genotype *P. vivax* VK210 was detected with a higher prevalence in *A. darlingi*. Interestingly, in the same State, in the vicinity of Rio Branco (636 km far from Cruzeiro do Sul), a study indicated *A. oswaldoi* as the main human malaria vector since it was infected in higher rate than *A. darlingi*^11^. This finding reinforces the need to monitor the circulation of the human malaria parasites and genotypes in anopheline mosquitoes in different areas of Brazilian Amazon.

About infection frequency, the *P. vivax* VK210 genotype was found widely distributed in Brazilian Amazon. This predominance may be related to its genetic diversity. Previous studies showed that *P. vivax* VK247 has just one subtype^23^, while *P. vivax* VK210 was classified in six different subtypes in Mexico and Nicaragua^24^ and seven in China^23^, suggesting that different polymorphisms may increase this variant adaptability to different geographical areas^24^.

In contrast to this distribution of VK210 genotype, there are regions where *P. vivax* VK247 predominates. In Colombia, for example, VK247 was more prevalent in certain regions due to an increase in the number of sporozoites in *A. albimanus* compared to mosquitoes infected with the VK210 genotype, suggesting that different vectors may carry different infections^25^. One hypothesis that reinforces this finding is that the VK210 genotype could be more immunogenic than VK247, which can induce the immune response against VK210 and consequently limit VK210 sporozoite production and trigger the favorable selection of VK247 sporozoites, which may result in a higher frequency of this genotype in some mosquito species^25^.

Concerning to the distribution of infection by plasmodia, *P. vivax* infection remains as the highest in the Brazilian Amazon (87.28% in 2018), and its prevalence by State studied here were: 78.58% in Acre state, 88.89% in Amapá, 91.29% in Pará and 91.44% in Roraima^3^. Following the timeline of human malaria *vivax* cases number and the distribution of *P. vivax* genotypes in mosquitoes, we hypothesize that these human cases were caused, mainly, by the VK210 genotype (see more in Supplementary Fig. S1). However, the genotype distribution profile has been changing overtime due to the gradual increase of *P. vivax* VK247 in Amapá, Pará and Roraima States. These findings can be reinforced for what has already been described on these genotypes distribution in Acre^11,12^, Amapá^7^, Pará^13,26–28^ and Roraima^5^.

Here, we demonstrate for the first time the genotype VK247 more frequent than VK210 in Roraima state, where *An. albitarsis* s.l is the main vector of human malaria. The first report of infection by *P. vivax* VK247 in *A. albitarsis* s.l. from Brazilian Amazon was from the municipality of Marabá (PA)^13^, and later, it was detected in the municipality of Goianésia do Pará (PA)^28^. These data, together to the fact that the frequency of this genotype in Pará state is increasing, indicate that this genotype may be adapting not only in this *Anopheles* species, but also in *A. darlingi*.

In addition to signs of adaptation of VK247 in mosquitoes, this variant also presented some changes in clinical profile in humans that may be related to its evolution. Other findings show that *P. vivax* VK247 is associated with higher parasite density levels in malaria patients in the Brazilian Amazon^28,29^, and the association with increased immune responses related to the disease’s clinical complications^29^, highlighting the importance of epidemiological studies in monitoring the process of adaptation of this genotype to mosquitoes.

The hypothesis that a particular CSP genotype can be adapted/disseminated among anopheline species can be reinforced by our research group data about the susceptibility of Brazilian Amazonian anopheline species to *P. vivax* CSP genotypes, such as *A. aquasalis* to the two genotypes^15^ and the detection of higher rate of VK247 infection (42.20%) when compared to VK210 (29.10%) in *A. darlingi*, whereas *A. nuneztovari* was more susceptible to VK210 (54.00%) than to VK247 (26.20%) (unpublished data).

Even with the limited availability of *P. vivax*-like monoclonal antibodies, previous studies have detected low frequency or absence of this genotype both in humans and mosquitoes in the Brazilian Amazon^12,25,28,30^. The distribution profile of mixed infections by CSP genotypes also showed changes over the years. In a study from 1996, it was detected a high number of mixed infections in the municipality of Belém, Pará state (69.60%) and only *P. vivax* VK210 as a single infection^26^. Almost 10 years later, another study reported an increase of single infections in Novo Repartimento, also in Pará state, with a slight reduction in the number of mixed infections^27^. More recently, in Goianésia do Pará, there was no detection of the three CSP variants together, showing few samples with mixed infections (24.5%) compared with single infections (75.50%)^28^. Although most of these studies have been done in humans, the same distribution profile is being found in anopheline mosquitoes in the most current periods, also indicating that these variants are adapting in such a way as to be found in the majority of cases as single infections, as in the case of the Pará state and other localities that had few mixed infections in mosquitoes.

The species *A. nuneztovari* s.l.*, A. oswaldoi, A. braziliensis* and *A. triannulatus* s.l., even at low frequencies, were found to be naturally infected by both *P. vivax* genotypes, VK210 and VK247. Other studies detected these mosquitoes transmitting other plasmodia species, confirming these anophelines as secondary vectors of human malaria in the Brazilian Amazon^7,11,13,31,32^. However, this condition must be analyzed with caution since it was showed that zoophilic anopheline mosquitoes can produce false positive results^33,34^.

Despite *P. vivax* VK210 remains the most prevalent genotype, detected mostly in *A. darlingi* mosquitoes followed by *A. albitarsis* s.l., the genotype VK247 showed signs of adaptation in the most current periods in these species. These evidences show the possible spread of this genotype in localities from Brazil. The differences in *Anopheles* infection and distribution highlight the importance of entomological studies for the control of human malaria. Considering the *falciparum* malaria elimination Brazilian plan that includes measures against the vector species, we believe that the spread and adaptation of *P. vivax* genotypes will be under control which will result on the human malaria cases diminution.

## Methods

### Study design

We conducted a retrospective descriptive study of anopheline distribution and infection by *P. vivax* CSP genotypes VK210 and VK247. Information about mosquito species, infection by *P. vivax* CSP, period of collection, and geographic coordinates of localities was obtained from epidemiological databases belonging to the Malaria and Entomology Laboratory of the Evandro Chagas Institute in Pará. We gathered data from four states of the Brazilian Amazon: Pará (PA) from 2000 to 2015, Amapá (AP) from 2000 to 2010, Roraima (RR) from 2000 to 2003 and from 2009 to 2011, and Acre (AC) from 2012 to 2015.

Data from 29 municipalities of Brazilian Amazon were considered (Supplementary Table S1), all of which had similar climate characteristics, with frequent rainfall, and hot and humid conditions with an annual average temperature of 26 °C^4^.

### Identification of anopheline mosquitoes and detection of CSP genotypes

*Anopheles* mosquitoes were captured in four hours (6–10 pm) or overnight (6 pm–6 am) collections on malaria transmission areas using human-landing catch (Supplementary Table S1). The 4 hours collections were performed by two specialized catcher and those of 12 hours by four professionals who took turns every three hours. All captures were conducted in peridomicile environment.

The mosquitoes were identified using entomological keys^22,35–37^. Anopheline species belonging to complexes of cryptic species were not differenced, considering only the expression sensu lato (s.l.). The detection of CSP genotypes in mosquitoes was done by ELISA on plates previously coated with anti-CSP monoclonal antibodies specific for *P. vivax* VK210, *P. vivax* VK247, *P. falciparum, and P. malariae*^38^.

### Statistical analysis

All data on mosquito collections were placed into Microsoft Excel for the calculation of the Sporozoite rate (SR) and Human biting rate (HBR) per year. The SR were calculated by the number of infected mosquitoes, divided by the total number of anopheline mosquitoes caught in the period^39^ and, HBR were calculated as the total number of anopheline mosquitoes caught landing on humans, divided by the number of catchers, divided by the number of hours spent sampling in the period^40^.

The abundance of anopheline mosquitoes and the number of genotypes infections in these mosquitoes were used to perform analyzes by Binomial test (two proportions) every two years, in order to verify differences overtime between the proportion of genotypes infection among the main species of infected mosquitoes. The significance level was set at 5% and using the software Bioestat^®^ 5.0^41^.

## Supplementary information

Supplementary information about Anopheles mosquites and P. vivax genotypes

## Data Availability

The datasets analyzed in this study may be solicited from the corresponding author and/or to Marinete Póvoa and Izis Sucupira researchers by request.
