# Supplementary material for: VK210 and VK247 genotypes of Plasmodium vivax in anopheline mosquitoes from Brazilian Amazon
Source: Sci Rep. 2019 Jun 28;9:9391. doi: 10.1038/s41598-019-45809-5 (PMC6599022; doi:10.1038/s41598-019-45809-5)
Supplement: Supplementary file 1 — Supplementary information about Anopheles mosquites and P. vivax genotypes [file 41598_2019_45809_MOESM1_ESM.pdf]

VK210 and VK247 genotypes of *Plasmodium vivax* in anopheline mosquitoes from Brazilian Amazon.

Erian de Almeida Santos[1,2*], Izis Mônica Carvalho Sucupira[2], Bruno Matheus de Oliveira Martins[2], Ricardo José de Paula Souza e Guimarães[3], Clístenes Pamplona Catete[3], Raimundo Tadeu Lessa de Souza[2], Ana Cecília Feio dos Santos[2], and Marinete Marins Póvoa[1,2]

[1]Federal University of Pará, Belém, Pará, 66075-110, Brazil.

[2]Evandro Chagas Institute, Laboratory of Basic Research in Malaria - EntomologyParasitology Section,Ananindeua, Pará, 67030-000, Brazil.

[3]Evandro Chagas Institute, Laboratory of Geoprocessing, Ananindeua, Pará, 67030-000, Brazil

[*]correspondingauthor. eriansantos.bio@gmail.com

Supplementary-Tables

Locality	Geographic coordinate	Characteristics of collections			
		Year	Season*	Collection time (No. of collections)	Overall hours of collection
**Pará state**					
Altamira	03º 12' 12" S, 52º 12' 23" W	2007	Rainy	4h (3)<br>12h (2)	36h
			Dry	12h (1)	12h
		2008	Rainy	4h (6)<br>12h (3)	60h
			Dry	4h (29)<br>12h (4)	164h
		2012	Rainy	4h (5)<br>12h (4)	68h
			Dry	4h (2)<br>12h (1)	20h
		2013	Rainy	4h (2)<br>12h (3)	44h
			Dry	12h (7)	84h
		2014	Rainy	12h (4)	48h
			Dry	12h (4)	96h
		2015	Rainy	12h (4)	48h
			Dry	4h (5)<br>12h (6)	92h
Anajás	00º 59' 12" S, 49º 56' 24" W	2002	Rainy	4h (10)	40h
Anapú	03º 28' 20" S, 51º 11' 52" W	2008	Rainy	4h (10)<br>12h (3)	76h
			Dry	4h (20)<br>12h (6)	152h
		2012	Rainy	4h (2)	8h
			Dry	4h (2)	8h
		2013	Rainy	4h (1)	4h
			Dry	4h (2)	8h
		2014	Rainy	4h (4)	16h
		2015		4h (2)	8h
Belém	01º 25’ S, 48º 77’ W	2000	Rainy	4h (11)<br>12h (1)	56h
			Dry	4h (10)	40h
		2001	Rainy	4h (7)	28h
			Dry	4h (6)	24h
		2003	Rainy	4h (1)	4h
			Dry	4h (10)	40h
		2004	Dry	4h (9)	36h
		2005	Rainy	4h (17)	68h
			Dry	4h (4)	16h

		2006	Rainy	4h (11)	44h
Canaã dos Carajás	06º 29' 49" S, 49º 52' 42" W	2010	Dry	4h (9)<br>12h (3)	72h
			Rainy	4h (7)	28h
		2012	Dry	4h (2)	8h
		2013	Rainy	12h (1)	12h
			Dry	12h (1)	12h
		2014	Rainy	12h (1)	12h
			Dry	12h (1)	12h
		2015	Rainy	4h (5)	20h
Goianésia do Pará	03º 50' 33" S, 49º 05' 49" W	2010	Dry	3h (4)<br>12h (2)	36h
		2011	Rainy	4h (4)<br>12h (2)	40h
			Dry	4h (3)<br>12h (4)	60h
		2012	Rainy	4h (6)<br>12h (6)	96h
			Dry	4h (6)<br>12h (2)	48h
Itaituba	04º 15’ S, 55º 59’ W	2003	Rainy	4h (1)<br>12h (1)	16h
			Dry	4h (2)<br>12h (1)	20h
		2011	Dry	4h (4)<br>12h (2)	40h
		2012	Rainy	4h (6)<br>12h (3)	60h
			Dry	4h (6)<br>12h (3)	60h
Jacundá	04º 27' 03" S, 49º 06' 59" W	2010	Dry	4h (4)<br>12h (1)	28h
		2012		3h (4)	12h
Juruti	02º 09' 08" S, 56º 05' 32" W	2006	Rainy	4h (7)	28h
			Dry	4h (8)<br>12h (3)	68h
		2007	Dry	4h (12)<br>12h (1)	60h
		2008	Rainy	4h (9)<br>12h (4)	84h
		2009	Dry	4h (7)<br>12h (3)	64h
		2010	Dry	4h (11)<br>12h (7)	128h
Marabá	05º 22' 07" S, 49º 07' 04" W	2005	Rainy	4h (4)	16h
		2006		4h (2)<br>12h (2)	32h
		2010	Dry	4h (2)<br>12h (1)	20h
		2012		4h (1)<br>12h (2)	28h
Moju	01º 52’ S, 48º 45’ W	2000	Dry	4h (19)	76h
		2010		4h (8)	28h
		2012		4h (5)	20h

Novo Progresso	07º 08' 52" S, 55º 22' 52" W	2003	Rainy	4h (4)	16h
			Dry	4h (1)<br>12h (4)	52h
Oriximiná	01º 45' 56" S, 55º 51' 58" W	2001	Dry	4h (6)	24h
		2002	Rainy	4h (4)<br>12h (1)	28h
			Dry	4h (6)<br>12h (2)	48h
		2003	Dry	4h (2)<br>12h (6)	80h
Pacajá	03º 50' 16" S, 50º 38' 15" W	2013	Dry	12h (4)	48h
		2014	Rainy	4h (2)	8h
			Dry	12h (2)	24h
		2015	Rainy	12h (2)	24h
			Dry	12h (2)	24h
Parauapebas	06º 04' 03" S, 49º 54' 08" W	2005	Rainy	4h (5)<br>12h (4)	68h
		2006	Rainy	4h (4)<br>12h (4)	64h
			Dry	4h (8)	32h
		2007	Rainy	4h (10)<br>12h (19)	268h
			Dry	4h (10)<br>12h (5)	100h
		2008	Rainy	4h (8)<br>12h (2)	56h
			Dry	4h (10)<br>12h (7)	124h
		2009	Dry	4h (6)<br>12h (2)	48h
		2010	Rainy	4h (4)	16h
		2012	Dry	4h (4)	16h
		2013	Rainy	4h (3)<br>12h (3)	48h
			Dry	12h (4)	48h
		2014	Rainy	4h (5)<br>12h (1)	32h
			Dry	4h (3)<br>12h (1)	24h
Santarém	02º 26' 35" S, 54º 42' 30" W	2002	Rainy	4h (4)	16h
			Dry	12h (1)	12h
		2003	Rainy	4h (4)	16h
			Dry	12h (4)	48h
		2004	Rainy	4h (4)	16h
		2005	Dry	12h (4)	48h
		2006	Dry	4h (4)	16h
São Caetano de Odivelas	00º 45' 00" S, 48º 01' 12" W	2000	Rainy	4h (1)	4h
			Dry	4h (1)	4h
		2001	Rainy	4h (16)	64h
			Dry	4h (13)	52h

		2002	Rainy	4h (7)	28h
Senador José Porfírio	02º 35' 27" S, 51º 57' 15" W	2003	Rainy	4h (16)<br>12h (5)	124h
			Dry	12h (2)	24h
		2007	Dry	12h (2)	24h
		2008	Rainy	12h (8)	96h
			Dry	12h (1)	12h
		2012	Rainy	12h (3)	36h
			Dry	4h (1)<br>12h (5)	64h
		2013	Rainy	12h (4)	48h
			Dry	12h (8)	96
		2014	Rainy	12h (2)	24h
			Dry	12h (8)	96h
		2015	Rainy	12h (3)	36h
			Dry	4h (1)<br>12h (3)	40h
Tucuruí	03º 45' 58" S, 49º 40' 21" W	2010	Dry	4h (1)<br>12h (2)	28h
		2012		4h (4)	16h
Viseu	01º 11' 48" S, 46º 08' 24" W	2000	Dry	4h (3)	12h
Vitória do Xingu	02º 52' 48" S, 52º 00' 36" W	2007	Dry	4h (4)<br>12h (2)	40h
		2008	Rainy	12h (1)	12h
			Dry	4h (15)<br>12h (4)	108h
		2012	Rainy	4h (7)<br>12h (2)	52h
			Dry	4h (15)<br>12h (10)	108h
		2013	Rainy	4h (4)<br>12h (4)	64h
			Dry	4h (6)<br>12h (9)	132h
		2014	Rainy	4h (4)<br>12h (5)	76h
			Dry	4h (6)<br>12h (10)	144h
		2015	Rainy	4h (2)<br>12h (5)	56h
			Dry	4h (7)<br>12h (6)	100h
**Amapá state**					
Macapá	00º 02' 20" N, 51º 03' 59" W	2000	Rainy	4h (5)<br>12h (6)	92h
			Dry	4h (5)	20h
		2001	Rainy	4h (13)	52h
			Dry	4h (19)	76h

		2002	Rainy	4h (7)	28h
			Dry	4h (18)	72h
		2003	Rainy	4h (2)<br>12h (1)	20h
			Dry	4h (4)<br>12h (3)	52h
		2004	Rainy	12h (2)	24h
			Dry	12h (2)	24h
		2005	Rainy	4h (11)<br>12h (2)	68h
			Dry	12h (4)	48h
		2006	Rainy	12h (5)	5/60h
		2007	Dry	12h (4)	48h
		2009		12h (4)	48h
		2010	Rainy	4h (1)<br>12h (1)	16h
Santana	00º 03' 30" S, 51º 10' 54" W	2000	Rainy	4h (2)<br>12h (2)	32h
			Dry	4h (3)	12h
		2001	Rainy	4h (7)	28h
			Dry	4h (2)	8h
		2002	Rainy	4h (3)	12h
			Dry	4h (4)	16h
		2005	Rainy	4h (2)	8h
			Dry	4h (2)	8h
		2007	Dry	4h (2)	8h
Serra do Navio	00º 53’44" N, 52º 00’08" W	2000	Dry	4h (5)	20h
		2001	Rainy	4h (5)	20h
Tartarugalzinho	01º 19’ N, 50º 57’ W	2001	Rainy	4h (5)	20h
**Roraima state**					
Boa Vista	02º 49’N, 60º 40’ W	2000	Rainy	4h (16)	64h
		2001	Rainy	4h (12)	48h
			Dry	4h (8)	32h
		2002	Rainy	4h (12)	48h
			Dry	4h (10)	40h
		2003	Rainy	4h (3)<br>12h (1)	24h
			Dry	4h (3)<br>12h (2)	36h
		2009	Dry	4h (2)<br>12h (1)	20h
		2011	Rainy	4h (3)<br>12h (1)	24h
Cantá	02º 36' 36" N,	2009	Dry	4h (1)	4h

	60º 35' 49" W				
Iracema	02º 10' 55" N, 61º 02' 28" W	2009	Dry	4h (3)<br>12h (2)	36h
		2011	Rainy	4h (2)<br>12h (2)	30h
**Acre state**					
Cruzeiro do Sul	07°37'51"S, 2°40'12"W	2012	Rainy	4h (6)<br>12h (1)	36h
			Dry	4h (5)<br>12h (2)	44h
		2013	Rainy	4h (4)<br>12h (1)	28h
			Dry	4h (17)<br>12h (2)	92h
		2014	Rainy	4h (2)<br>12h (2)	32h
			Dry	4h (5)<br>12h (3)	56h
		2015	Rainy	4h (6)<br>12h (1)	36h
			Dry	4h (5)<br>12h (3)	56h

Supplementary Table S1.Localities and informations about collections from Brazilian Amazon, 2000-2015. *December to May correspond to rainy season and June to November to Dry season.

Locality	No. of mosquitoes collected	Total of CSP infection (%)	CSP Infection		Sporozoite rate (%)		HBR
Pará state			VK210	VK247	VK210	VK247	
2000							
A. darlingi	1,071	6 (0.56)	6	-	0.56	-	2.85
A. aquasalis	336	6 (1.79)	6	-	1.79	-	0.89
A. nuneztovaris.l.	689	10 (1.45)	4	6	0.58	0.87	1.83
Total	2,561	22 (0.86)	16	6	0.62	0.23	6.81
2002							
A. darlingi	7,381	47 (0.64)	45	2	0.61	0.03	21.46
*A. oswaldoi*s.l.	165	3 (1.82)	-	3	-	1.82	0.48
A. nuneztovaris.l.	747	3 (0.40)	3	-	0.40	-	2.17
Total	9,663	53 (0.55)	48	5	0.50	0.05	28.09
2003							
A. darlingi	2,003	22 (1.10)	17	5	0.85	0.25	2.28
*A. albitarsis*s.l.	2,235	36 (1.61)	31	5	1.39	0.22	2.54
A. nuneztovaris.l.	261	1 (0.38)	1	-	0.38	-	0.30
Total	4,769	59 (1.24)	49	10	1.03	0.21	5.42
2004							
A. darlingi	798	44 (5.51)	33	11	4.13	1.38	7.67
Total	800	44 (5.50)	33	11	4.12	1.37	7.69
2005							
A. darlingi	128	7 (5.47)	3	4	2.34	3.12	0.30
*A. albitarsis*s.l.	562	8 (1.42)	7	1	1.25	0.18	1.30
*A. oswaldoi*s.l.	37	1 (2.70)	1	-	2.70	-	0.09
Total	766	16 (2.09)	11	5	1.44	0.65	1.77
2006							
A. darlingi	56	2 (3.57)	2	-	3.57	-	0.10
*A. albitarsis*s.l.	2,289	2 (0.09)	2	-	0.09	-	4.03
Total	3,018	4 (0.13)	4	-	0.13	-	5.31
2007							
A. darlingi	292	6 (2.05)	4	2	1.37	0.68	0.27
*A. albitarsis*s.l.	449	5 (1.11)	5	-	1.11	-	0.42
Total	1,644	11 (0.67)	9	2	0.55	0.12	1.52
2008							
A. darlingi	4,726	36 (0.76)	25	11	0.53	0.23	2.50
*A. albitarsis*s.l.	1,048	6 (0.57)	6	-	0.57	-	0.56
A. nuneztovaris.l.	214	2 (0.93)	2	-	0.93	-	0.11
*A. triannulatus*s.l.	155	2 (1.29)	-	2	-	1.29	0.08
Total	6,274	46 (0.73)	33	13	0.53	0.21	3.32
2010							
A. darlingi	35	1 (2.86)	-	1	-	2.86	0.04
*A. albitarsis*s.l.	532	6 (1.13)	3	3	0.56	0.56	0.68
Total	1,494	7 (0.47)	3	4	0.20	0.27	1.91
2011							
A. darlingi	103	3 (2.91)	2	1	1.94	0.97	0.37
Total	118	3 (2.54)	2	1	1.69	0.85	0.42
2012							
A. darlingi	2,793	56 (2.01)	39	17	1.40	0.61	1.74
*A. albitarsis*s.l.	1,225	15 (1.22)	-	15	-	1.22	0.76
A. nuneztovaris.l.	170	2 (1.18)	2	-	1.18	-	0.11
Total	6,072	73 (1.20)	41	32	0.67	0.53	3.78
2013							

A. darlingi	232	13 (5.60)	7	6	3.02	2.59	0.18
A. nuneztovaris.l.	186	3 (1.61)	2	1	1.07	0.54	0.14
Total	880	16 (1.82)	9	7	1.02	0.79	0.68
Amapá state							
2000							
A. darlingi	480	2 (0.42)	2	-	0.42	-	1.36
*A. albitarsis*s.l.	2,443	24 (0.98)	23	1	0.94	0.04	6.94
Total	3,280	26 (0.79)	25	1	0.76	0.03	0.93
2001							
A. darlingi	2,805	12 (0.43)	12	-	0.43	-	6.88
*A. albitarsis*s.l.	3,289	34 (1.03)	30	4	0.91	0.12	8.06
A. braziliensis	597	2 (0.34)	2	-	0.33	-	1.46
Total	7,190	48 (0.67)	44	4	0.61	0.06	1.76
2002							
A. darlingi	737	23 (3.12)	21	2	2.85	0.27	2.88
*A. albitarsis*s.l.	2,260	47 (2.08)	36	11	1.59	0.49	0.88
Total	3,906	70 (1.79)	57	13	1.46	0.33	15.26
2003							
A. darlingi	264	1 (0.38)	1	-	0.38	-	1.83
*A. albitarsis*s.l.	935	32 (3.42)	16	16	1.71	1.71	6.49
Total	1,283	33 (2.57)	17	16	1.32	1.25	8.91
2004							
A. darlingi	213	2 (0.94)	1	1	0.47	0.47	2.22
*A. albitarsis*s.l.	716	28 (3.91)	21	7	2.93	0.98	7.46
Total	931	30 (3.22)	22	8	2.36	0.86	9.70
2005							
A. darlingi	347	9 (2.59)	8	1	2.30	0.29	1.31
*A. albitarsis*s.l.	2,245	36 (1.60)	33	3	1.47	0.13	8.50
Total	2,649	45 (1.70)	41	4	1.55	0.15	10.03
2006							
A. darlingi	227	12 (5.29)	5	7	2.20	3.08	1.89
*A. albitarsis*s.l.	343	19 (5.54)	10	9	2.91	2.62	2.86
Total	695	31 (4.46)	15	16	2.16	2.30	5.79
Roraima state							
2000							
A. darlingi	144	3 (2.08)	3	-	2.08	-	1.13
*A. albitarsis*s.l.	539	7 (1.30)	7	-	1.30	-	4.21
Total	936	10 (1.07)	10	-	1.07	-	7.31
2001							
A. darlingi	233	8 (3.43)	8	-	3.43	-	1.46
*A. albitarsis*s.l.	2,698	52 (1.93)	46	6	1.70	0.22	16.86
Total	3,559	60 (1.69)	54	6	1.52	0.17	22.24
2002							
A. darlingi	876	15 (1.71)	12	3	1.37	0.34	4.98
*A. albitarsis*s.l.	7,690	67 (0.87)	67	-	0.87	-	4.37
A. braziliensis	328	9 (2.74)	9	-	2.74	-	1.86
Total	8,967	91 (1.01)	88	3	0.98	0.03	50.95
2003							
A. darlingi	195	3 (1.54)	3	-	1.54	-	1.63
*A. albitarsis*s.l.	1,014	10 (0.99)	4	6	0.39	0.59	8.45
Total	1,209	13 (1.08)	7	6	0.58	0.50	10.08
2009							
A. darlingi	125	2 (1.60)	1	1	0.80	0.80	1.04
*A. albitarsis*s.l.	201	2 (1.00)	-	2	-	0.99	1.68
Total	868	4 (0.46)	1	3	0.11	0.35	7.23

2011							
*A. albitarsis*s.l.	384	8 (2.08)	2	6	0.52	1.56	3.43
Total	599	8 (1.34)	2	6	0.33	1.00	5.35
Acre state							
2012							
A. darlingi	584	17 (2.91)	15	2	2.57	0.34	3.65
Total	589	17 (2.89)	15	2	2.55	0.34	3.68
2013							
A. darlingi	1,956	33 (1.69)	28	5	1.43	0.26	8.15
Total	1,958	33 (1.69)	28	5	1.43	0.25	8.16
2014							
A. darlingi	938	7 (0.75)	5	2	0.53	0.21	5.33
Total	938	7 (0.75)	5	2	0.53	0.21	5.33
2015							
A. darlingi	895	10 (1.12)	8	2	0.89	0.22	4.86
*A. albitarsis*s.l.	1	1 (100.00)	1	-	100.00	-	0.01
Total	897	11 (1.23)	9	2	1.00	0.22	4.88

Supplementary Table S2. Data on mosquitoes naturally infected by *P. vivax* CSP genotypes, Brazilian Amazon, 2000 to 2015.

Supplementary figure

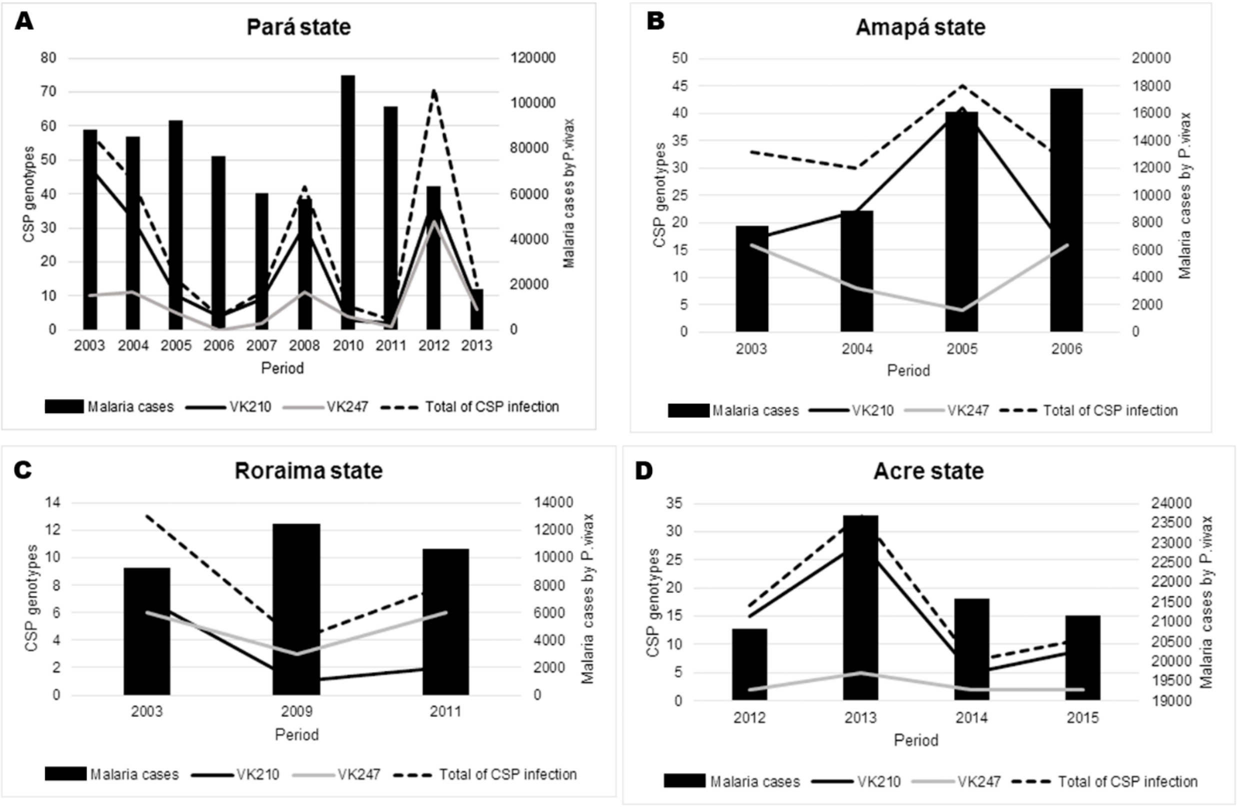

Supplementary Figure S1. Number of malaria cases and *P. vivax* CSP genotypes in (A) Pará, (B) Amapá, (C) Roraima and, (D) Acre states, 2003 to 2015.
